# Poly (I:C)- and doxorubicin-loaded magnetic dendrimeric nanoparticles affect the apoptosis-related gene expressions in MCF-7 cells

**DOI:** 10.3906/biy-1912-71

**Published:** 2020-08-19

**Authors:** Rouhollah KHODADUST, Aktan ALPSOY, Gözde ÜNSOY, Ufuk GÜNDÜZ

**Affiliations:** 1 Department of Biotechnology, Middle East Technical University, Ankara Turkey; 2 Department of Biological Sciences, Middle East Technical University, Ankara Turkey; 3 Department of Biotechnology, Hamidiye Health Science Institute, University of Health Science-Turkey, İstanbul Turkey

**Keywords:** Targeted drug delivery, magnetic dendrimeric nanoparticles, Poly(I:C), doxorubicin, gene expression

## Abstract

Use of nanoparticles as drug carrier vectors has great potential to circumvent the limitations associated with chemotherapy, including drug resistance and destructive side effects. For this purpose, magnetic generation 4 dendrimeric nanoparticles were prepared to carry chemotherapeutic agent doxorubicin (G_4_-DOX) and immune modulator polyinosinic:polycytidylic acid [Poly(I:C)]. As previously reported, DOX and Poly(I:C) was loaded onto G_4_ nanoparticles (PIC-G_4_-DOX). Cellular internalization study using confocal microscopy demonstrated high levels of cellular internalization of PIC-G_4_-DOX nanoparticles by MCF-7 cells. This resulted in higher efficacy of PIC-G_4_-DOX nanoparticles in killing MCF-7 breast cancer cells. Alteration in the expression levels of selected genes was determined by RT-qPCR analyses. Proapoptotic NOXA, PUMA, and BAX genes were upregulated, and SURVIVIN, APOLLON, and BCL-2 genes were downregulated, indicating the cell-killing effectiveness of PIC-G_4_-DOX nanoparticles. Gene expression analysis provided some insights into the possible molecular mechanisms on cytotoxicity of DOX and Poly(I:C) delivered through G_4_ magnetic nanoparticles. The results demonstrated that PIC-G_4_-DOX can be useful for targeted delivery affecting apoptotic pathways, resulting in an advanced degree of cancer-cell–killing. They are promising for targeting cancer-cells because of their stability, biocompatibility, higher internalization, and toxicity.

## 1. Introduction 

Nanotechnology-based delivery vehicles, such as nanoparticles, nanocapsules, micelles, or dendrimers, have great potential to be utilized in nanomedicine to deliver conventional drugs, recombinant proteins, vaccines, and more recently genetic material, by addressing the problems related to poor solubility, high toxicity, nonspecific delivery, in vivo degradation, and short circulation half-lives of conventional drugs, which often limits optimal dosage at the target site (Bansal et al., 2020). 

Magnetic nanoparticles are popular for many biological applications, including targeted drug delivery and magnetic hyperthermia, in which they can be directed to a tumor site under a magnetic field. Superparamagnetic iron oxide nanoparticles, which align in the presence of a magnetic field, are mostly preferred for the targeted delivery of drugs and diagnostics agents. In order to obtain biocompatible, multifunctional magnetic nanoparticles, their surfaces can be modified with different organic and inorganic polymers (Ling et al., 2019).

Dendrimers are hyperbranched globular polymeric nanostructures with a unique tree-like architecture and compact spherical geometry in solution. Dendrimers can be prepared at various generations, which involves the multiple layers of branched structures. They are utilized in targeted drug delivery with their nano size, narrow polydispersity index, controlled molecular structure, and multiple functional group availability (Svenson and Tomalia, 2012). Chemotherapeutic drugs or nucleic acids such as siRNA, DNA/RNA vaccines, and targeting moieties are conjugated to the terminal functional groups of dendrimers which act as nonviral transfection agents in gene therapy (Palmerston et al., 2017). Dendrimers are suitable candidates for the delivery of nucleic acids attributed to structural flexibility and hyperbranched architectures (Pedziwiatr-Werbicka et al., 2013). While the net charge of dendrimers is positive under physiological conditions, it is possible to form complexes with nucleic acids. This complex can then bind to negatively charged surface molecules on a cell membrane. Followed by internalization of nanoparticles into the cells via endocytosis, release of nucleic acids takes place (Parker-Esquivel et al., 2012).

Polyamidoamine (PAMAM) dendrimers are 3-dimensional branched polymers used in gene delivery. The highly branched structure maximizes surface area and exposes surface functional groups for interactions, making the dendrimers suitable for binding a variety of molecules (Kaur et al., 2016). The positively charged terminal amines of PAMAM dendrimers are thought to bind to the negatively charged phosphate backbone groups of the nucleic acid, facilitating an electrostatic interaction between the dendrimers and nucleic acid. The higher generation dendrimers are more positively charged than the lower generations (Abedi-Gaballu et al., 2018). 

Doxorubicin (DOX) is an anthracycline antibiotic which inhibits DNA and RNA synthesis in mammalian cells as an effective anticancer agent. It can efficiently accumulate in the nucleus, intercalate with DNA, and act as a cytostatic and apoptotic agent in cancer cells. The production of free radicals and oxidative stress is highly involved in the toxic and anticancer mechanisms of DOX (Kim and Kim, 1972). The clinical application of DOX has some limitations due to its serious side effects (cardiotoxicity, heart damage) and the development of resistance (Singal et al., 2000). DOX-loaded nanoparticles has been used to overcome these limitations. DOX was loaded into the cavities of synthesized dendrimer-coated magnetic nanoparticles. Loading on PAMAM dendrimers enhances the aqueous solubility and bioavailability of DOX, and ensures its controlled release (Chanphai et al., 2017). Moreover, nanoparticle-mediated delivery of DOX copes with the drug resistance via bypassing Pgp-mediated efflux pumps (Da Silva et al., 2017)

Poly (I:C) is a synthetic dsRNA which has been well characterized. The structure of Poly (I:C) is similar to viral dsRNA and boosts the immune response by activating endosomal toll-like receptor 3 (TLR3) or cytosolic melanoma differentiation-associated protein 5 (MDA-5) and retinoic acid-inducible gene I (RIG-1) receptors. It is a potent vaccine adjuvant and apoptotic agent which stimulates TLR3 and activates interferons against pathogens and cancer cells. Poly (I:C) improves the efficacy of chemotherapeutic drugs when used concurrently in clinical trials. However, Poly (I:C) cannot be taken up into the cells by itself (Yoneyama et al., 2004)

Dendrimer was used to load DOX and attach Poly (I:C) onto the magnetic nanoparticles (Khodadust et al., 2014). PAMAM dendrimers form complexes with nucleic acids, protect Poly (I:C) from nuclease degradation, and are considered cell-penetrating molecules. After the cell penetration of the dendrimeric nanoparticles, Poly (I:C) binds to the TLR3 receptors and activates apoptotic pathways. The toxicity of the cationic dendrimers increases with increasing generations; however, multiple studies have demonstrated that generation 4 PAMAM dendrimers (G_4_), such as the one used in this study, are nontoxic up to 250 µg/mL concentrations and effectively deliver nucleic acids into the cells (Khodadust et al., 2013). In vitro analyses have also proved that the higher generations of cationic PAMAM dendrimers induce aggregation of human platelets in plasma proportional to the number of surface amines (Salvador et al., 2012)

Previously in our laboratory, different generations of dendrimer-coated magnetic nanoparticles (DcMNPs) were synthesized, characterized, and successfully used for the delivery of DOX and codelivery of Poly (I:C) and DOX (Khodadust et al., 2014). These formulations were convenient for delivering anticancer agents into the breast cancer cells, resulting in effective killing of cancer cells. However, their effect on proapoptotic and antiapoptotic signaling pathways involving changes in gene expression levels is a matter for concern. Sustained change in the expression of apoptotic family proteins (antiapototic Bcl-2-like and proapototic subgroups) are very important in the survival and development of different cancer types. Therefore, the targeting of cellular antiapoptotic proteins or application of proapoptotic proteins as therapeutic modalities have emerged as key targets to be implemented in cancer therapy (An et al., 2019). In this study, quantitative PCR (qPCR) analyses were employed to investigate the effects of drug-loaded/-unloaded G_4_ MNPs on apoptotic and antiapoptotic gene expressions of MCF-7 cells. For this purpose, antiapoptotic BCL-2, SURVIVIN, and APOLLON and proapoptotic BAX, NOXA, and PUMA genes were chosen. 

## 2. Materials and methods

### 2.1. Materials

Ferric chloride hexahydrate (FeCl_3_6·H_2_O), ferrous chloride tetrahydrate (FeCl_2_4·H_2_O), ammonia solution (NH3) (32%), 3-aminopropyl trimethoxysilane (APTS) [NH_2_ (CH_2_)_3_-Si-(OCH_3_)_3_], methylacrylate, methanol, ethanol and ethylenediamine, phosphate buffer saline (PBS), ethylenediaminetetraacetic acid, trisaminomethane, 1-ethyl-3-(3-dimethylaminopropyl) carbodiimide hydrochloride (EDC), polyinosinic–polycytidylic acid (Poly [I:C]), and acetic acid were purchased from Sigma Aldrich (St. Louis, MO, USA) and were used in the synthesis of PAMAM-coated MNPs. The 2,3-bis(2-methoxy-4-nitro-5-sulfophenyl)-5-((phenylamino)carbonyl)-2H-tetrazoliumhydroxide (XTT) reagent was purchased from Biological Industries (Kibbutz Beit-Haemek, Israel). DOX was kindly provided by Gulhane Military Academy (School of Medicine, Ankara, Turkey). Human serum was donated by the authors of this manuscript (Rouhollah Khodadust and Pelin Mutlu) for physiological stability studies. 

### 2.2. DOX loading and Poly (I:C) binding on G_4_ magnetic dendrimeric nanoparticles

Poly (I:C)-bound DOX-loaded magnetic dendrimeric nanoparticles (PIC-G_4_-DOX) were improved to obtain an efficient drug delivery system as explained in our previous studies (Khodadust et al., 2014). Briefly, DOX was loaded on G_4_ with 96% drug entrapment. Poly (I:C) was then bound covalently onto the surface of G_4_-DOX nanoparticles through the 1-ethyl-3-(3-dimethylaminopropyl) carbodiimide hydrochloride (EDC) activation method (Sheehan et al., 1961). Poly (I:C) loading on G4 was previously optimized, and the ratio of Poly (I:C) to nanoparticles was reported as 1:10 (w/w) by our group (Khodadust et al., 2014). 

### 2.3. Cellular internalization of PIC-G_4_-DOX

MCF-7 cells were grown on cover glasses placed in 12-well tissue culture plates in complete media. On the following day, cells were incubated with G_4_-DOX solution for 3 h. After the incubation, old medium was removed from the plates and the cells were washed 3 times with PBS. DAPI staining was performed according to the manufacturer’s instructions. For this purpose, 300 µM DAPI intermediate dilution was prepared by adding 2.1 µL of the 14.3 mM DAPI stock solution to 100 µL PBS. Then 300-µM DAPI intermediate solution diluted as 1:1000 in PBS was added as needed to make a 300-nM DAPI stain solution. Next, 500 µL of the diluted DAPI staining solution was added to the wells, incubated for 5 min, and washed 2–3 times with PBS. The cells were visualized with a Zeiss confocal laser scanning microscope (Zeiss LSM 700; Carl Zeiss Microscopy GmbH, Oberkochen, Germany). The LSM picture files were analyzed using Image J software. 

### 2.4. Cytotoxicity of PIC-G_4_-DOX

Cytotoxicity of the free PIC, PIC-G_4_, and DOX solutions at various concentrations was determined using the XTT cell proliferation assay as described previously. According to these results, the IC50 values of free PIC, PIC-G_4_, and DOX were calculated as 450 µg/mL (PIC), 28 µg/mL (PIC), and 170 µg/mL for free DOX, respectively (Khodadust et al., 2014).

### 2.5. Expression of apoptosis-related genes 

MCF-7 cells (300,000 cells per well) were seeded in a 6-well plate. After overnight incubation, cells were treated with G_4_DcMNP, G_4_-DOX, PIC-G_4_-DOX, and free DOX. According to the literature, about 18 h of treatment with the required concentration of DOX could be enough to see the drug’s toxicity effect at the mRNA level (Asnani et al., 2018; Alessandrini el., 2018). Therefore, cells were treated with 18µg/mL, 6µg/mL, and 0.6µg/mL of each nanoparticle formulation for 18 h. After incubation, cells were collected with Tri Pure RNA isolation reagent (Roche Diagnostics GmbH, Mannheim, Germany) and stored at –80 °C until used. RNA purity and quantity were determined using a Nanodrop spectrometer (Thermo Scientific, Waltham, MA, USA). Next, 1 mg RNA was treated with DNase I (Thermo Scientific), which was then inactivated at 65 °C in the presence of EDTA. RNA was then converted to cDNA using random hexamer and Revert Aid reverse transcriptase (Thermo Scientific). Primers were designed and verified with Primer3-web (http://primer3.ut.ee/) and Primer blast (http://www.ncbi.nlm.nih.gov/tools/primer-blast/) programs, respectively (Table).

**Table:** Primer sequences of β-actin, SURVIVIN, APOLLON, BCL-2, NOXA, PUMA and BAX genes.

Primer	Sequences
Β-actin sense	5’CCAACCGCGAGAAGATGA3’
Β-actin antisense	5’CCAGAGGCGTACAGGGATAG3’
SURVIVIN sense	5’AGCCAGATGACGACCCATAGAGG3’
SURVIVIN antisense	5’AAAGGAAAGCGCAACCGGACGA3’
APOLLON sense	5’TAGTGATATGCCTCGTTTGTTGG3’
APOLLON antisense	5’TTCTGTGTGTGCTCACCTTTC3’
BCL-2 sense	5’TGTGGCCTTCTTTGAGTTC3’
BCL-2 antisense	5’CGGTTCAGGTACTCAGTCATC3’
NOXA sense	5’TGATATCCAAACTCTTCTGC3’
NOXA antisense	5’ACCTTCACATTCCTCTCAA3’
PUMA sense	5’GACGACCTCAACGCACAGTA3’
PUMA antisense	5’GTAAGGGCAGGAGTCCCAT3’
BAX sense	5’TCTGACGGCAACTTCAACTG3’
BAX antisense	5’TTGAGGAGTCTCACCCAACC3’

All of the amplicons were amplified using SYBR Green Master Mix (Roche) with reaction conditions as 95 °C for 10 min enzyme activation, 40 cycles of 95 °C for 15 sec, and 60 °C for 1 min. RT-qPCR results were analyzed with the 2^-DDCt^ method (Livak and Schmittgen, 2001). Fold changes are expressed relative to untreated MCF-7 cells.

## 3. Results and discussion

### 3.1. DOX loading and Poly (I:C) binding on G_4_

**Figure 1:**
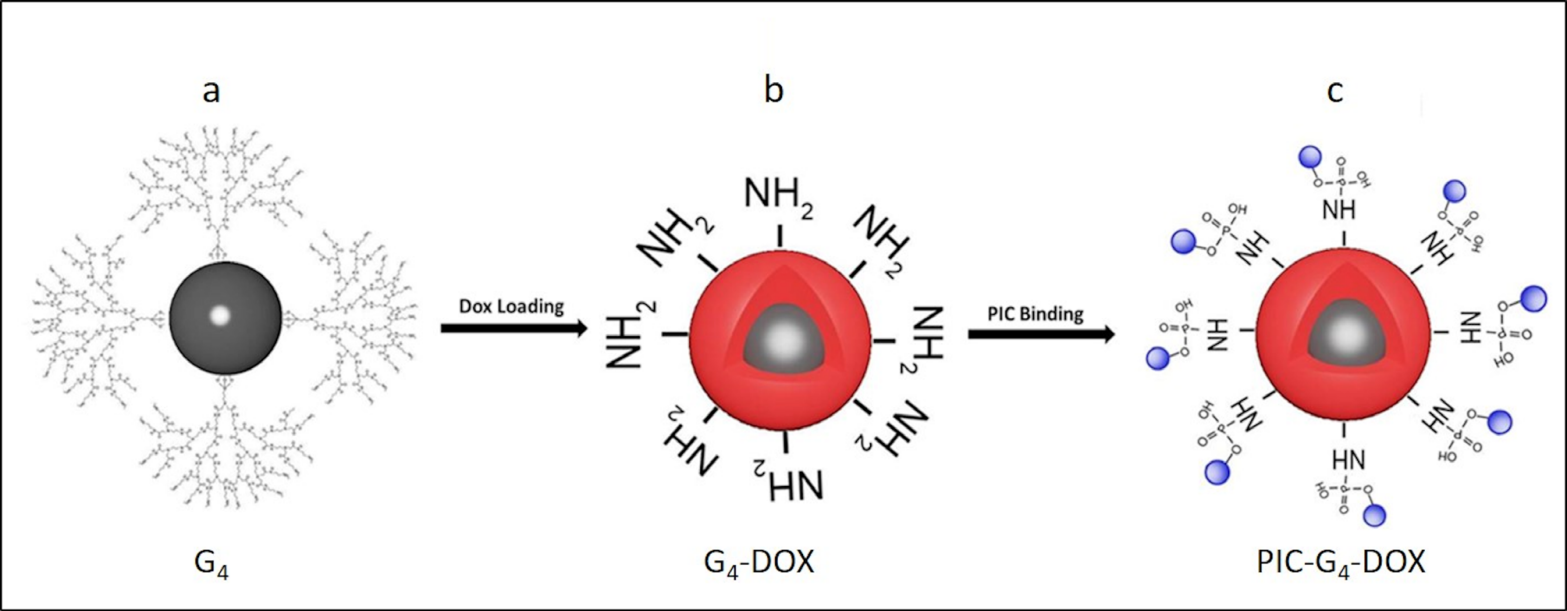
Schematic representation of magnetic generation 4 dendrimeric nanoparticles (G_4_) (a), chemotherapeutic agent doxorubicin- loaded (the red layer) nanoparticles (G_4_-DOX) (b) and immune modulator polyinosinic:polycytidylic acid Poly(I:C) bound nanoparticles (PIC-G_4_-DOX) (c).

The surface of the G_4_-PAMAM dendrimer resembles pockets. At neutral pH, an electrostatic repulsion happens at the surface of the dendrimer owing to the positive charge of the primary amines, which itself results in the formation of a number of cavities (Martiniano et al., 2020). Here, fourth-generation PAMAM dendrimers (G_4_) were synthesized and loaded with DOX (Figure 1a–b). The drug content, drug entrapment, and drug loading percentages of G_4_ were identified as 13%, 96%, and 100%, respectively. The equations for the calculations are given (Equations 1–3). 

Equation 1:$\mathrm{Drug Content}(\%,\frac{w}{w})=\frac{(\mathrm{mass of total drug}-\mathrm{Mass of free Dryg}}{\mathrm{Mass of nanoparticles}}x100$

Equation 2: $\mathrm{Drug entrapment}(\%,\frac{w}{w})=\frac{(\mathrm{mass of total drug}-\mathrm{Mass of free Dryg}}{\mathrm{Mass of total drug}}x100$

Equation 3 $\mathrm{Drug loading}(\%,\frac{w}{w})=\frac{(\mathrm{mass of total drug}-\mathrm{Mass of free Dryg}}{\mathrm{Mass of total polymer}}x100$

**Figure 2:**
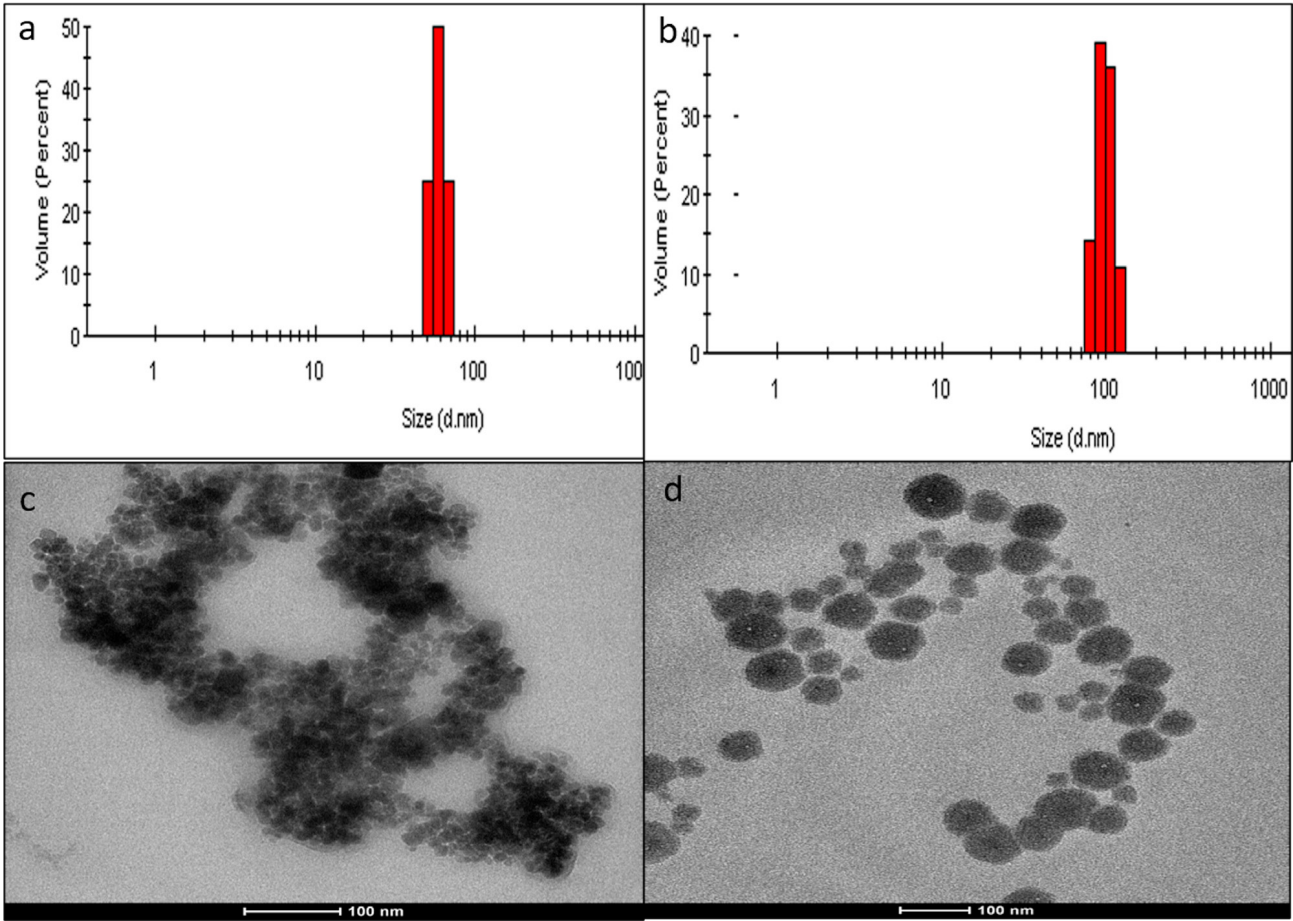
DLS results of G_4_DcMNPs (a) and PIC-G_4_-DOX (b). TEM images of G_4_DcMNPs (c) and PIC-G_4_-DOX (d). The scale bar in the TEM images shows 100 nm.

Poly (I:C) was covalently bound onto the surface of G_4_-DOX in the presence of EDC reagent and imidazole at pH 6.5 (Figure 1c). Since Poly (I:C) covers the surface of dendrimers, binding Poly (I:C) in advance makes the DOX unable to enter the cavities of the dendrimeric nanoparticles. For this reason, DOX was loaded into the cavities of G4 before Poly (I:C) binding. Loading DOX into the cavities of nanoparticles in advance allows us to overcome the steric hindrance at the dendrimer surface and improves the stability and reactivity of NPs (Khandare et al., 2005). The distance between the surface functional groups increased with the increased amounts of DOX loaded inside the cavities of G_4_. This improved the binding efficiency of Poly (I:C). Accordingly, the surface functional groups became 10 times more responsive to Poly (I:C) binding when the DOX was loaded into the cavities of G4 (Khodadust et al., 2014). According to the DLS results, after DOX loading and Poly (I:C) modification, the size of DcMNPs increased from 60 ±15 nm to 100 ±25 nm. Comparison of TEM results of DcMNPs and PIC-G4-DOX demonstrated that doxorubicin loading together with Poly (I:C) modification improves the dispersivity of the complex (Figure 2). 

### 3.2. Cellular internalization of G_4_-DOX

**Figure 3:**
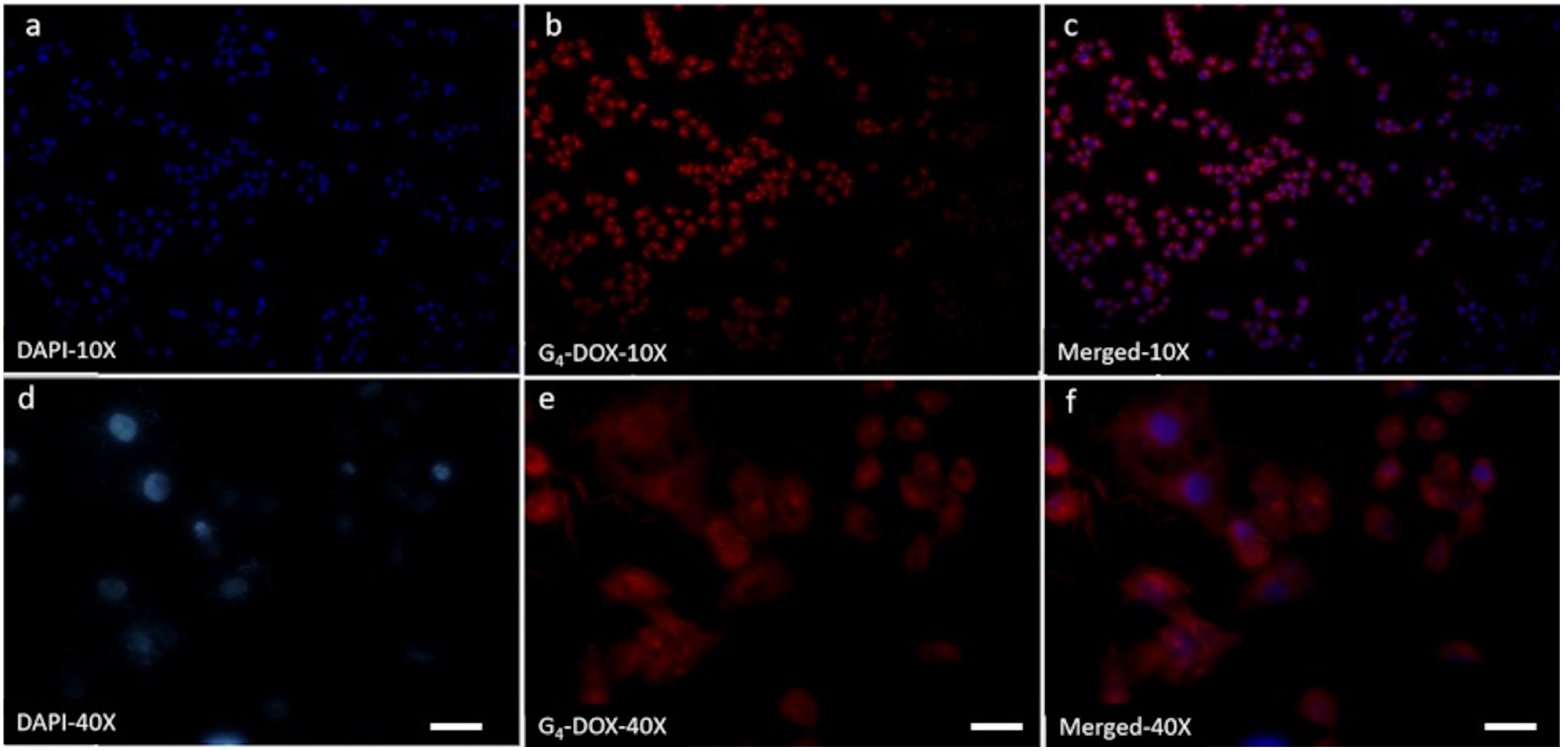
Cellular uptake images of G_4_-DOX internalized with MCF-7 cells at 10x and 40x resolution. Visualization of DAPI nuclear labeled G_4_-DOX treated cells with DAPI filter (a and b, 10x and 40x, respectively). Visualization of G_4_-DOX treated cells with red filter (excitation 543 nm, emission longpass filter 570 nm) sutiable for doxorubicin (c and d, 10x and 40x, respectively). Merged images of DOX (red) and nuclei (blue) in G_4_-DOX treated cells (e and f, 10x and 40x, respectively).

In order to specify the cellular internalization of DOX loaded nanoparticles, breast cancer cells were incubated with G_4_-DOX for 3 h. After cell fixation and nuclear staining with DAPI, the images of the cells were obtained by confocal laser scanning microscope with 2 different objectives, allowing different regions of cells to be viewed. Confocal laser scanning microscope images of G_4_-DOX nanoparticle-treated cells with DAPI filter (Figures 3a and 3b) and red filter (suitable for DOX at 543 nm excitation and 570 nm emission longpass filter) (Figures 3c and 3d), together with merged images, demonstrated that the drug-loaded nanoparticles were internalized into all cells (Figures 3e and 3f). 

Free DOX can pass through the cell membrane by diffusion, which is driven by a concentration gradient. Due to its hydrophobic anthracycline backbone, cellular membrane is highly permeable to DOX (Yacoub et al., 2011). Therefore, free DOX passes through the cell membrane; some of it enters the nucleus, where it inhibits DNA replication (Sui et al., 2014). 

Although free DOX passes easily through the cell membrane, its hydrophobic structure is a disadvantage for solubility in aqueous media. Another disadvantage of DOX application is its harsh cardiotoxic side effects (Singal et al., 2000). The development of drug resistance mechanisms restricts free DOX application in cancer chemotherapy (Mirski et al., 1987). On the other hand, DOX-loaded G_4_ (G_4_-DOX) is taken up into the cells by endocytosis, which bypasses the drug resistance mechanisms. DOX is released from G_4_-DOX nanoparticles in a controlled manner inside the acidic endosomes and is diffused into the cytosol before it finally enters the cell nucleus (Khodadust et al., 2013).

### 3.3. Cytotoxicity of PIC-G_4_-DOX

Our group previously demonstrated that G_4_ and G_7_ do not show any significant cytotoxicity in MCF-7 cells at concentrations up to 250 µg/mL and 120 µg/mL, respectively (Khodadust et al., 2014). Moreover, loading of 5 µg/mL Poly (I:C) on 80 µg/mL G_7_ nanoparticles results in 30% cell death. However, the surface functional groups of G_7_ can be efficiently bound with therapeutic agents such as Poly (I:C); their rigid surface structures do not allow other drugs to enter the cavities of nanoparticles. On the other hand, the surface structures of G_4_ are more flexible than G_7_, which allows drugs to enter the cavities. Thus, the lower generations of dendrimers are more suitable for drug loading and delivery with their lower toxicity. DOX was loaded into the cavities of G_4_. This helps to overcome steric hindrance at the surface and makes the surface functional groups more appropriate for the binding of therapeutic agents such as Poly (I:C) (Khodadust et al., 2014). Poly (I:C) was then bound onto the surface of G_4_-DOX through EDC activation. Its binding onto the surface functional amine groups of dendrimers also improves the biocompatibility of the nanoparticles by decreasing the cationic load (Khandare et al., 2005).

Our previous results indicated that the codelivery of DOX and Poly (I:C) on G4 are more effective in killing cancer cells than the delivery of either agent alone (Khodadust et al., 2014). Such additive or synergistic effect is not that obvious with G_7_ due to the low amounts of DOX loaded. Lower generations of dendrimer-coated magnetic nanoparticles with their internal cavities and longer circulation time in blood are more suitable for drug delivery applications (Khodadust et al., 2014).

### 3.4. Expression of apoptosis-related genes 

The apoptotic signaling pathway is regulated by a group of complex molecules in a network, involving the expression changes of distinct proapoptotic and antiapoptotic genes. In cancer cells, apoptosis can be induced either by the activation of upstream molecules of apoptosis pathway or by the inhibition of antiapoptotic factors.

**Figure 4:**
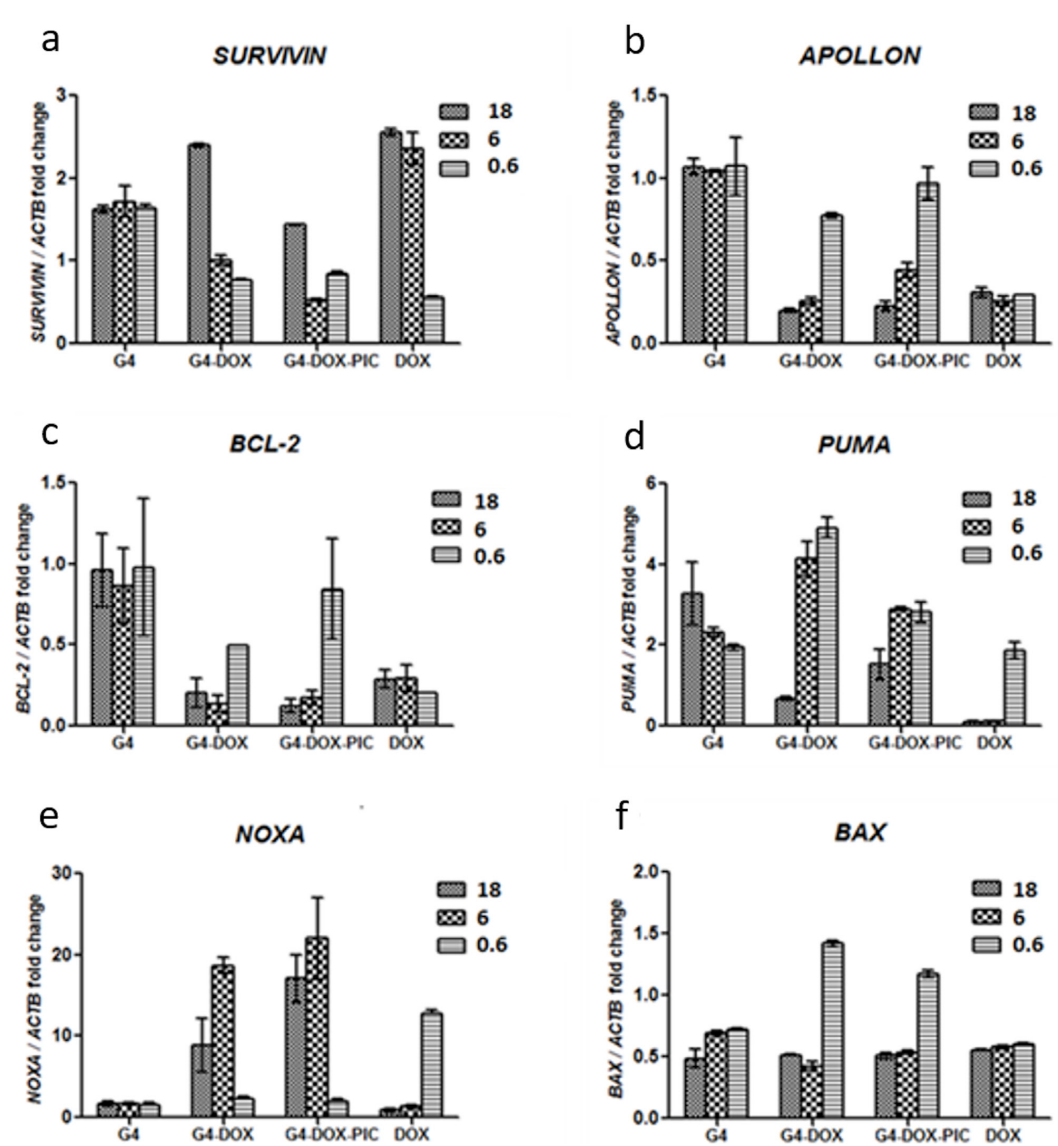
Expression analysis of different classes of apoptosis-related genes: SURVIVIN (a), APOLLON (b), BCL-2 (c), PUMA (d) NOXA (e) and BAX (f). MCF-7 cells were treated with bare nanoparticles (G_4_), DOX-loaded G_4_ nanoparticles (G_4_-DOX) and Poly (I:C) modified-DOX loaded G4 nanoparticles (G_4_-DOX-PIC) and free DOX at different doses (18 μg/mL, 6 μg/mL, 0.6 μg/mL doxorubicin equivalent amounts of nanoparticles) for 18 h.

DOX increases the production of reactive oxygen species (ROS) and stimulates protein degradation in the cell with the caspase-3 and ubiquitin–proteasome pathway. Application of free DOX, G_4_, G_4_-DOX, and PIC-G_4_-DOX changed the expression levels of certain genes, including SURVIVIN*, *APOLLON, and BCL-2 (antiapoptotic proteins), as well as NOXA, PUMA, and BAX (major regulators of the intrinsic apoptotic pathway) (Figure 4). 

SURVIVIN, a member of the inhibitor of apoptosis protein (IAP) family, is a caspase blocker that selectively inhibits the intrinsic apoptosis pathway. SURVIVIN expression is a determinant of DOX response in breast cancer cells (Faversani et al., 2014). It has also been reported that P-gp–mediated resistance to DOX is modulated by SURVIVIN (Faversani et al., 2014). We showed that SURVIVIN expression was upregulated 2-fold at high doses of free DOX (18 µg/mL and 6 µg/mL), while delivery of DOX in G4 with Poly (I:C) (PIC-G4-DOX) or alone (G_4_-DOX) reduced the expression of SURVIVIN at all 3 doses (18 µg/mL, 6 µg/mL, and 0.6 µg/mL) compared to G_4_ (Figure 4a). Codelivery of DOX and Poly (I:C) in G_4_ could prevent SURVIVIN induction, which may be the potential mechanism for increased cytotoxicity compared to free DOX. It has been demonstrated that Poly (I:C) downregulates the expression of antiapoptotic proteins, especially SURVIVIN and BCL-XL in HCC cells, which is compatible with our findings (Yuan et al., 2008). However, there are other reports that imply independence of DOX response from SURVIVIN status. According to De Moraes’s (2013) study, SURVIVIN expression was reduced by 80% after treatment with 5mM DOX in MCF-7 cells for 24 h (De Moraes et al., 2013), which is contradictory to our results. SURVIVIN overexpression does not cause resistance to DOX in MCF-7 cells (Liu et al., 2007).

The silencing of the APOLLON gene, an inhibitor of apoptosis protein (IAP), can lead to increased apoptosis in various breast cancer cell lines by activating caspases 3, 8, and 9 (Yoneda et al., 2008). Unlike other IAPs such as SURVIVIN, XIAP (De Moraes et al., 2013; Liu et al., 2007), siRNA-mediated knockdown of APOLLON had more pronounced cytotoxic effect on MCF-7 cells (Yoneda et al., 2008). Knockdown of the APOLLON gene has also been reported to enhance chemosensitivity to a cisplatin/docetaxel regimen (Zhang et al., 2014). We observed that both G_4_-DOX and G_4_-PIC-DOX caused dose-dependent downregulation in APOLLON expression (Figure 4b), while APOLLON expression in cells treated with free DOX remained in around 30% of untreated cells in all doses. This is the first report of DOX causing APOLLON downregulation in breast cancer cells.

BCL-2 is one of the major antiapoptotic members of the BCL-2 family involved in the intrinsic apoptotic pathway. It has been shown that BCL-2 was downregulated in MCF-7 cells in response to DOX (Chorna et al., 2005) and that BCL-2 overexpression conferred resistance to DOX in MCF-7 cells (Zhang et al., 2014). Here we observed that G_4_-DOX and PIC-G_4_-DOX downregulates BCL-2 slightly more than free DOX at higher doses (6 and 18 mg/mL). We did not see a significantly dose-dependent response in BCL-2 expression after the application of free DOX (Figure 4c). Previously it was shown that Poly I:C treatment downregulates BCL-2 in dose-dependent manner in human umbilical vein endothelial cells (HUVECs) (Sun et al., 2011). However, we did not observe either an additive or a synergistic effect between Poly I:C and DOX in BCL-2 levels on MCF-7 cells. 

PUMA is a BH3-only proapoptotic protein that is generally induced by p53. The BH3 (BCL-2 Homology 3) domain is referred to as the “death domain” and is the minimum requirement for a protein to have proapoptotic functions. Previous reports showed that the effect of DOX treatment on PUMA expression was highly context-dependent, where in some cells PUMA expression remained constant while in others it gradually increased first and then decreased. In 6 h of DOX treatment, PUMA expression increased in a dose-dependent manner in MCF-7 cells (Dudgeon et al., 2012). Our results showed that 6 and 18 mg/mL of free DOX downregulated PUMA by 10-fold while the lowest dose displayed 2-fold upregulation (Figure 4d). This could be explained by the observation that PUMA expression may gradually increase and then decrease upon various DOX treatments, where an 18-h treatment might fall in the “decline” phase. When DOX was delivered via nanoparticles as G_4_-DOX and PIC-G_4_-DOX, PUMA expression was inversely correlated with the dose. This was compatible with the results obtained with treatment of free DOX. Therefore, we hypothesized that since the bioavailability of DOX and exposure to high-dose DOX are not as high as free DOX in treatments with nanoparticles due to the gradual release, the downregulation phase of PUMA expression would have come later. Thus, PUMA expression levels might have started to decline in 18 h compared to an equivalent dose of free DOX, which might enable a longer period of high PUMA expression in G_4_ nanoparticle-treated cells. 

NOXA is another proapoptotic protein induced by p53. NOXA is upregulated by DOX treatment in various breast cancer and neuroblastoma cell lines (Dudgeon et al., 2012). In addition, Poly I:C treatment upregulated NOXA in a time-dependent manner in human umbilical vein endothelial cells (HUVECs) (Davis et al., 2003). We demonstrated that G_4_-DOX and PIC-G_4_-DOX upregulated NOXA (10-fold to 20-fold), the 6 mg/mL dose being the highest NOXA inducer in both cases. At the lowest dose, neither formulation induced NOXA at a significant level (Figure 4e). At the highest doses of free DOX, NOXA expression remained approximately the same as untreated MCF-7 cells, while it was 12-fold upregulated by the lowest dose of DOX. Based on these observations, we could suggest that NOXA could follow a similar expression pattern to PUMA under DOX treatment. In addition, the data suggested that there might be a contribution of Poly I:C in NOXA expression. It was observed that in 18 h at both 6 and 18 mg/mL, PIC-G_4_-DOX induced NOXA more efficiently than G_4_-DOX at the corresponding doses. These results validate that the DOX routes cells to apoptosis with the aid of PUMA and NOXA gene expressions.

BAX is a proapoptotic BCL-2 family member of the major intrinsic apoptotic pathway. It has been previously shown that DOX induces BAX expression in MCF-7 cells (Davis et al., 2003). However, our results displayed that free DOX downregulated BAX expression by half in all 3 doses (Figure 4f). We observed approximately 3- and 2-fold upregulation of BAX levels at 0.6 mg/mL G_4_-DOX and PIC-G_4_-DOX treatments, respectively, relative to G_4_. In addition, empty G_4_ nanoparticles reduced BAX expression by half, reflecting their nontoxicity.

Vivek et el. (2014) investigated the possible apoptotic signaling pathways of “smart” pH-responsive chitosan nanoparticles for controlled release of oxaliplatin. The transcription levels of antiapoptotic SURVIVIN, BCL-2, BAX, and Bik proteins are important in the induction of apoptosis. The potential apoptotic mechanisms between oxaliplatin and oxaliplatin-loaded chitosan nanoparticle-treated MCF-7 cells were investigated at the protein level by Western blot analyses. Oxaliplatin-loaded chitosan nanoparticles significantly upregulated the protein levels of BAX, Bik, Cytochrome C, caspase-9 and -3, and downregulated the antiapoptotic proteins such as BCL-2 and SURVIVIN. The antiapoptotic protein BCL-2 mainly inhibits the mitochondrial pathways, while SURVIVIN directly blocks the processing and activation of effectors caspase-3 and caspase-9, which commonly act downstream of apoptosis signaling pathways (R Vivek et al., 2013), which suggests that oxaliplatin loaded with chitosan nanoparticles induced apoptosis of MCF-7 cells through the intrinsic apoptotic signaling pathway (Raju Vivek et al., 2014).

Chen et al. studied a novel DOX delivery system based on titanium dioxide (TiO_2_) nanoparticles to enhance the anticancer efficacy of a drug while reducing its side effects. They explored the changes in the expression levels of apoptosis-regulating proteins including BAX, BCL-2, and caspase 3 by Western blot, and showed that DOX-loaded TiO2 nanoparticles are more efficient in BAX induction and *BCL-2 *repression compared to free DOX in a human SMMC-7721 hepatocellular carcinoma cell line (Chen et al., 2011). Upregulated BAX leads to the disruption of mitochondrial membrane integrity, resulting in caspase-3 activation and apoptosis (Ghavami et al., 2009).

**Figure 5:**
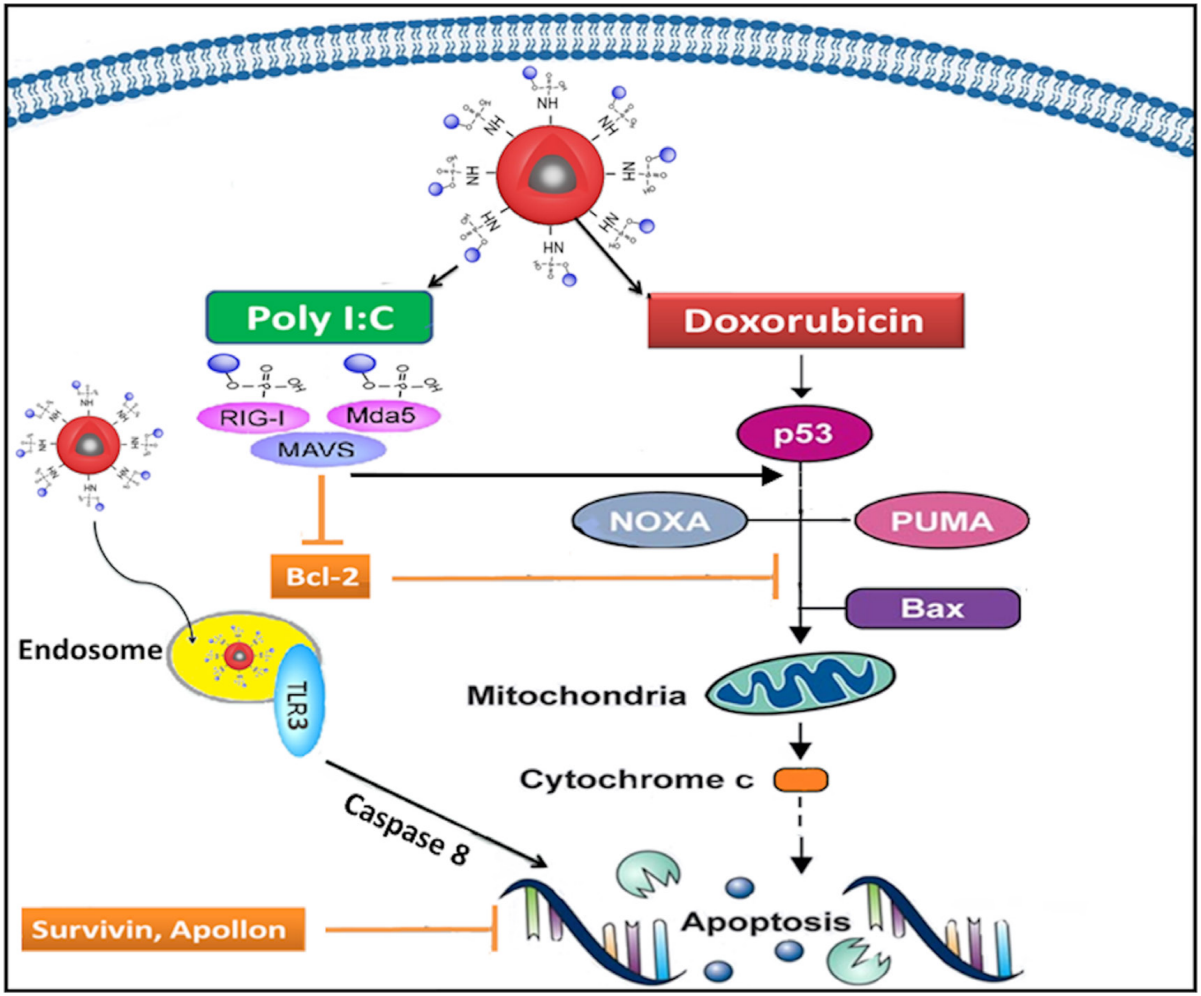
Simplified schematic representation for the molecular pathway of Poly (I:C) bound DOX loaded DcMNPs (PIC-G_4_-DOX) on MCF-7 cells.

The possible roles of these apoptosis-related genes of interest inside the cells after the internalization of DOX and Poly I:C carrier G_4_ nanoparticles are schematized in Figure 5. Poly I:C behaves like an RNA virus and induces the intrinsic apoptosis pathway. After receptor-mediated endocytosis, the synthetic dsRNA in the cytoplasm is sensed by a class of ubiquitous cytoplasmic RNA helicases, retinoic acid inducible gene-I (RIG-I) (Yoneyama et al., 2004), and melanoma differentiation Ag-5 (MDA-5) (Kato et al., 2006), which trigger an antiviral signaling cascade via the adaptor protein mitochondrial antiviral signaling (MAVS) (also called IFN-b stimulator 1 [IPS-1]), virus-induced signaling adapter (VISA), caspase activation, and recruitment domain adaptor inducing IFN-b (Cardif) (Kawai et al., 2005; Seth, Sun, Ea, and Chen, 2005) of the intrinsic apoptotic pathway (Vince and Tschopp., 2010).

As depicted in Figure 5, activation of p53 from stress stimuli leads to the activation of BAX by upregulating PUMA and NOXA, and finally initiates the intrinsic apoptosis pathway. After activation, proapoptotic BAX leads to the depolarization of mitochondria and release of cytochrome c into the cytosol. Cytochrome c makes a complex with apoptosis protease activation factor (APAF-1) to form an apoptosome, then binds to pro-caspase 9. Activated caspase 9 cleaves the effector caspase 3, which is responsible for executing apoptosis. Thus, BAX is an important factor required for mitochondrial outer membrane permeabilization (MOMP) and the subsequent release of cytochrome c (Strasser et al., 2011). Effective MOMP depends on so-called BH3-only proteins such as NOXA and PUMA that sense the apoptotic stimulus. 

MAVS-dependent signaling results in the activation of the transcription factors Nuclear Factor kappa B (NF-kB) and interferon regulatory factor (IRFs), such as IRF-3, which can act separately to induce inflammatory cytokines and chemokines or act together as part of an enhanceosome to induce type I interferons (IFNs). Activation of RIG-I and subsequent downstream MAVS signaling can induce transcription of the proapoptotic BH3-only proteins NOXA and PUMA independent of p53, resulting in activation of the intrinsic apoptotic pathway, whereby BAX functions to disrupt mitochondrial membrane integrity leading to cytochrome C release, formation of apoptosome, caspase 3 activation, and apoptosis (Figure 5) (Besch et al., 2009). 

## 4. Conclusion

In conclusion, we have reported on the fourth generation (G_4_) of magnetic dendrimeric nanoparticles as a drug delivery system. Furthermore, applicability of these nanoparticles as a carrier for the delivery of the cationic anticancer drug DOX and Poly (I:C) was demonstrated in vitro. Codelivery of DOX and Poly (I:C) on G4 were more effective in killing cancer cells than the delivery of either agent alone. Enhanced cytotoxicity of a drug when loaded on nanoparticles can be attributed to the higher internalization of nanoparticles as compared to the free drug. Although the same amounts of free DOX were applied, higher efficacy is obtained with G_4_ since higher amounts of nanoparticles enter the cells. This shows the important role of G_4_ dendrimeric nanoparticles as an efficient carrier for the delivery of anticancer drugs. Furthermore, death mechanisms of MCF-7 breast cancer cells cultured with DOX and Poly (I:C) carrier G_4_ nanoparticles have been investigated with the RT-qPCR method. The expression levels of certain proapoptotic (NOXA, PUMA, and BAX) and antiapoptotic genes (SURVIVIN, APOLLON, BCL-2) have changed in accordance with the amount of drug-loaded nanoparticles. Synthesized nanoparticles have exhibited superior cancer-cell–killing potential since they are internalized at high amounts by the cells and alter the pro- and antiapoptotic pathways. Development of magnetic dendrimeric nanocarriers that can target tumors and release of drugs at specific tumor sites represents a valuable solution for overcoming drug resistance, minimization of side effects, and effective antitumor therapy.
